# Content validation of the Wound-QoL questionnaire measuring quality of life in chronic wounds – a qualitative study in patients with leg ulcers and diabetic foot ulcers

**DOI:** 10.1186/s41687-025-00935-9

**Published:** 2025-10-22

**Authors:** Toni Maria Janke, Beke Hester, Julia Jahn, Judith Rusch, Matthias Augustin, Franziska Zirkenbach, Ewa Klara Stürmer, Christine Blome

**Affiliations:** 1 Institute for Health Services Research in Dermatology and Nursing, University Medical Center Hamburg-Eppendorf, Martinistraße 52, 20246 Hamburg, Germany; 2 Department of Vascular Medicine, University Heart and Vascular Center, University Medical Center Hamburg-Eppendorf, Hamburg, Germany

**Keywords:** Chronic wounds, Content validity, Health-related quality of life, Patient-reported outcomes measures, Wound-QoL

## Abstract

**Background:**

The Wound-QoL-17 and its short version, the Wound-QoL-14, measure health-related quality of life in patients with chronic wounds. This study assessed the content validity of this questionnaire.

**Methodology:**

We recruited adult patients with chronic wounds in outpatient and inpatient settings in Germany. We conducted semi-structured interviews, which were audio-recorded, transcribed verbatim and analysed using qualitative content analysis.

**Results:**

Almost all of the 21 patients (mean age 63 years, *n* = 16 male) had leg ulcers (*n* = 11) or diabetic foot ulcers (*n* = 8). The analysis resulted in six main categories: items; relevance; comprehensibility; comprehensiveness; version Wound-QoL-17 vs. Wound-QoL-14; further aspects. Participants mostly understood the distinct items well and found them easy to answer and relevant to their situation. The overall questionnaire was mostly rated relevant, comprehensible and comprehensive, including instructions, response scale, and recall period.

**Conclusions:**

This study confirms the content validity of the Wound-QoL for patients with leg ulcers or diabetic foot ulcers and shows that it adequately reflects the construct of wound-specific quality of life. The Wound-QoL-17 should be used in clinical settings where differentiated assessment is appropriate. In research contexts where the calculation of scores is paramount, the Wound-QoL-14 should be used.

**Supplementary Information:**

The online version contains supplementary material available at 10.1186/s41687-025-00935-9.

## Background

Wounds are considered chronic if they have not healed for four to eight weeks after they first occurred [1] or when they are caused by an underlying condition or cause, such as venous insufficiency, peripheral arterial occlusive disease, diabetes or constant pressure [2]. The estimated prevalence of chronic wounds is 1.67 per 1,000 persons [3] with higher prevalence rates in older people [4], which make this condition especially relevant in ageing societies. Chronic wounds present not only an economic burden on societal level [4], but also individual burden for patients [5] and informal caregivers [6, 7]. Patients’ lives might be impacted by time-consuming treatment [5], burdensome comorbid conditions [8] and restrictions in their health-related quality of life (HRQoL) [4]. HRQoL is a multidimensional construct describing well-being and functionality of a person, which is influenced by the health and illness as well as the treatment of illness [9]. Chronic wounds cause impacts on various dimensions of HRQoL. This includes physical burden caused by exudate, odour and wound pain, as well as psychological strain leading to sleep disturbances, anxiety and depression [10]. Living with chronic wounds can restrict patients’ everyday life activities, social activities and financial status [11, 12].

The Wound-QoL-17 was developed in 2014 to measure HRQoL in patients with chronic wounds [11, 12]. The Wound-QoL-17 is based on three previously existing instruments: the Freiburg Life Quality Assessment for Wounds (FLQA-w [13]), the Cardiff Wound Impact Schedule (CWIS [14]), and the Würzburg Wound Score (WWS [15]). As these three instruments are either relatively long or do not provide the possibility to calculate subscale scores, the goal in developing the Wound-QoL was to establish a short instrument which can be easily used in clinical practice and also enables evaluation of different domains of HRQoL [11]. As a basis for the development of the Wound-QoL-17, patients filled in all three instruments (FLQA-w, CWIS and WWS); using qualitative and quantitative measures, the most important items and content were selected. An expert group then defined the items for the Wound-QoL-17. The final Wound-QoL-17 contains 17 items reflecting the three subscales body, psyche and everyday life, while one item on financial burden is only part of the global scale. Each item is rated on a five-point Likert scale with higher values indicating higher impairments. Psychometric properties of the Wound-QoL-17 have been tested for various language versions (e.g [16–23]). Later on, a shorter version, the Wound-QoL-14, was developed and validated [24–26]. The questionnaires can be used to evaluate HRQoL in patients with chronic wounds in both clinical care and research.

However, throughout the development process of the Wound-QoL, no content validation with patients was conducted. According to the Consensus-based Standards for the selection of health Measurement Instruments (COSMIN), content validity is the most important measurement property of a patient-reported outcome measure (PROM) [27]. It is the degree to which a questionnaire adequately reflects the construct to be measured and comprises relevance, comprehensiveness and comprehensibility of the PROM regarding the construct, the target population and the context of use of interest [27].

Therefore, this study aimed to assess the content validity of the original version of the Wound-QoL, the Wound-QoL-17, through qualitative interviews with patients. Specifically, we aimed to find out how patients understand the content of the items, whether they find the items relevant, easy to understand and comprehensible, and whether they think that the questionnaire comprehensively covers relevant aspects of their wound-related HRQoL.

## Methods

The study was approved by the Ethics Commission of the corresponding medical center. All participants provided written informed consent. This article follows the consolidated criteria for reporting qualitative research (COREQ) checklist.

### Interview guide and interviews

**Fig. 1:**
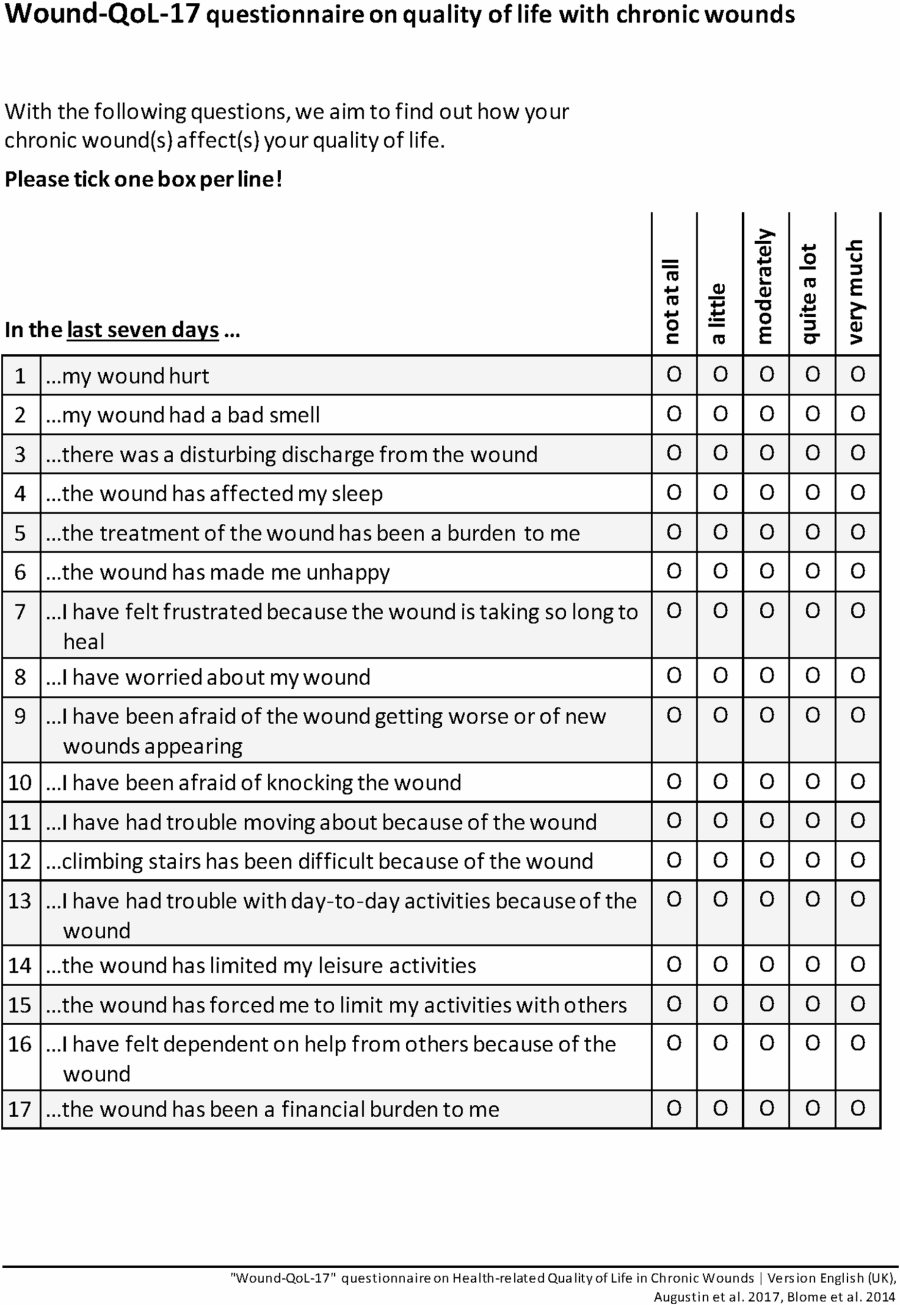
UK-English translation of the Wound-QoL-17

Semi-structured, guideline-based in-person interviews were conducted with patients with chronic wounds. The interview guide was developed by one of the authors based on the COSMIN guidelines [28] and discussed and revised with two researchers experienced in qualitative research. As an icebreaker question, patients were asked about their experiences with chronic wounds and the wound that they currently have. Afterwards, we used the think-aloud method [28, 29]: while completing the paper version of the Wound-QoL-17 (see Fig. 1 for English translation), patients verbalised their thoughts. If this had not yet been discussed, they were then asked specifically about the relevance and comprehensibility of each item as well as the relevance, comprehensibility and comprehensiveness of the questionnaire also including instructions, response options and recall period. The interview guide was pilot tested with four patients, which indicated that no changes were required. The full interview guide can be found in Appendix A1. All interviews were conducted in person by one author (JJ), who is a female psychology student in her final year of the master’s degree, who had previously conducted interviews for another study and who is trained in patient conversation. For this study, she received feedback from a qualitatively experienced researcher after the first interviews were completed. The interviewer had no personal or professional relationship with the study participants.

### Recruitment and sample

We included adult patients diagnosed with venous or arterial leg ulcer (LU), diabetic foot ulcer (DFU) or pressure ulcer (PU) at the time of the interview. The wound had to have existed for at least four weeks. Furthermore, patients had to be cognitively able to complete an interview lasting approximately 30 min. The only exclusion criterion for participation were wounds caused by autoimmune processes. Patients were approached by medical staff and informed about the study. They were recruited consecutively via the dermatological outpatient clinic and a wound center at a large hospital in Germany. The interviews were conducted in German referring to the original German version of the Wound-QoL questionnaire. At these facilities, interviews were conducted face-to-face with only interviewer and interviewee being present.

Based on the COSMIN criteria, we aimed to include seven to ten patients per diagnostic group; however, ultimately, the saturation of data determined the number of interviews [28], which was documented on an ongoing basis.

All interviews were audio-recorded and transcribed verbatim. In the process of transcription, the data were pseudonymised. No field notes were included in the analyses.

### Analysis

All interviews were analysed through qualitative content analysis according to Mayring [30], while taking a critical realist approach to ontology assuming that patient statements reflect their emotions, cognitions, and beliefs [31]. Lower hierarchical categories were derived inductively based on participants’ responses and then grouped into deductively developed categories, which were based on the questionnaire items and the aspects of relevance, comprehensibility and comprehensiveness. The first half of the interviews were coded in parallel by two researchers independently. Differences and unclarities were then discussed and one researcher continued to code all remaining interviews. The established category system was frequently discussed with further researchers. For the analyses, the software MAXQDA (Version 2022, VERBI Software, Berlin, Germany) was used.

## Results

We invited 25 patients to participate in this study of which 21 took part in interviews. Reasons for refusal were time constraints or not feeling fit enough. Three interviews were shortened or deviated slightly from the interview guide (duration 13 to 24 min) because participants were not able to read the questionnaire as they did not have their glasses, had experienced chronic wounds only in an inpatient setting, or were not able to complete the full interview due to consequences of another illness. The other interviews lasted between 29 to 80 min.

### Patient characteristics

The mean age of participants was 63 years and 16 were male. The most common diagnosis was LU (*n* = 11). More detailed information on patient characteristics can be found in Table 1.

**Table 1:** Patient characteristics

Characteristics		Participants (*N* = 21) Mean (Range) / *n* (%)
Age (years)		63.2 (33–79)
Gender	Female	5 (24%)
	Male	16 (76%)
Highest school education	Higher education (12 or 13 years)	9 (43%)
	Middle school (10 years)	6 (29%)
	General education (9 years)	5 (24%)
	No qualification	1 (5%)
Diagnosis	Venous leg ulcer	6 (29%)
	Arterial leg ulcer	5 (24%)
	Diabetic foot ulcer	8 (38%)
	Pressure ulcer	2 (10%)
Living situation	Alone	8 (38%)
	In community (e.g., with spouse/family)	10 (48%)
	Unknown	3 (14%)
Accessibility of home	Use of stairs necessary	9 (43%)
	No stairs present	8 (38%)
	Unknown	4 (19%)
Wound-QoL-17 score		1.53 (0.07–3.18)

### Category system

The category system we developed consists of six main categories with up to three hierarchical levels of subcategories. The six main categories are as follows:

- **Items**: Includes a subcategory for each item of the Wound-QoL-17. Each of these subcategories was further subcategorised including item content, relevance, comprehensibility and comprehensiveness.
- **Questionnaire relevance**: Describes the relevance of the overall questionnaire.
- **Questionnaire comprehensibility**: Includes statements on whether patients generally understood the items, the instructions, the recall period and the response options.
- **Questionnaire comprehensiveness**: Includes subcategories regarding the scope of the Wound-QoL, suggestions for merging individual items, items that patients thought were redundant, as well as aspects that patients thought were missing.
- **Version Wound-QoL-14 vs. Wound-QoL-17**: Covers patients’ responses regarding whether they think that the three items that are left out in the short version are relevant or not.
- **Further aspects**: Includes information on patients’ way of handling their wound as well as overall suggestions for improvement of the Wound-QoL.

The category system was almost identical for patients with LU and patients with DFU. Accordingly, results found for the whole sample can be applied for both patient groups. Patients with PU are included in the analysis of the total sample, but no conclusion can be drawn specifically for this subgroup due to its small size.

### Items

In the following, the results on all items are presented. It can be assumed that the results of the items can be applied to the corresponding subscales. If comprehensibility and comprehensiveness are not reported here, these aspects were rated as given. The number of participants rating each item as relevant and not relevant according to the statements in the interviews is depicted in Table 2.

**Table 2:** Patients’ rating of relevance of items (German questionnaire used in the study; wording presented below are from the official english (UK) translation of the Wound-QoL)

Item No.	Item wording	Relevance rating	
	In the last seven days …	Relevant	Not relevant
1	…my wound hurt.	14	5
2	…my wound had a bad smell.	8	11
3	…there was a disturbing discharge from the wound.	10	6
4	…the wound has affected my sleep.	10	7
5	…the treatment of the wound has been a burden to me.	10	6
6	…the wound has made me unhappy.	9	6
7	…I have felt frustrated because the wound is taking so long to heal.	14	3
8	…I have worried about my wound.	14	0
9	…I have been afraid of the wound getting worse or of new wounds appearing.	13	3
10	…I have been afraid of knocking the wound.	10	10
11	…I have had trouble moving about because of the wound.	11	6
12	…climbing stairs has been difficult because of the wound.	7	6
13	…I have had trouble with day-to-day activities because of the wound.	10	6
14	…the wound has limited my leisure activities.	11	4
15	…the wound has forced me to limit my activities with others.	7	2
16	…I have felt dependent on help from others because of the wound.	12	5
17	…the wound has been a financial burden to me.	7	11

#### Item 1 (Pain)

Participants considered variability of pain in intensity and sensation (“*I could say that in the worst case it was a bit itchy*”, DFU-07, male, 42 years). They also mentioned the impact on sleep (“*Tonight I had quite a lot of pain in the wound. That is*,* when the heat gets to it and the duvet over it/ I walked around for an hour.*”, LU-08, female, 77 years) as well as the impact on mobility. Some patients reported that pain was mostly present during or after wound care. The item was assumed to be relevant for both HRQoL and treatment. As pain varies over time, some patients thought it was difficult to give a suitable answer on the Likert scale, as they, for example, tried to form an average value: “*But then the pain was / sometimes more*,* sometimes less and that’s why I say moderately.*” (LU-09, female, 75 years).

#### Item 2 (Odour)

Wound odour was perceived as an indicator of deterioration and was mainly perceptible during changes of wound dressings. Some patients stated to find it unpleasant to expose the odour to others such as the wound care specialist. While some patients stated that “*that’s an important question.*” (LU-06, male, 79 years), others did not consider it as relevant as their “*wound never really smelled. It was never that important.*” (LU-01, female, 42 years).

#### Item 3 (Exudate)

Exudate led to uncertainty about the wound situation as patients assumed that it indicates delayed healing or deterioration: “*[…] you constantly have wound discharge somewhere*,* that is doesn’t heal or something*,* you do worry about that*,* right.*” (DFU-03, male, 56 years). Additionally, exudate led to increased duration and frequency of dressing changes. It also impacted patients’ choice of clothing as they reported not to wear shorts so that others do not see their leaking wound.

#### Item 4 (Sleep)

The item on sleep was often associated with impairment due to pain. Patients also mentioned problems positioning the affected limb (“*But I couldn’t get my legs up […] and also with the wound dressings*,* after short time they began to sag and then the pain was even worse.*”, LU-05, female, 54 years) and issues handling the tube of the drainage at night.

#### Item 5 (Treatment)

The term treatment was primarily associated with treatment by medical staff (“*For me*,* treatment always means that a doctor is involved. And not*,* just not the daily routine.*”, LU-03, male, 56 years). While some patients referred only to treatment in hospital or at the physician’s practice, others also considered wound care at home. Many patients did not find the treatment itself most burdensome but the time restrictions related to it: “*I had to think a bit at first*,* does it mean the actual wound care*,* or does it also mean all around it?*” (LU-04, male, 72 years); “*You’re constantly organising and rescheduling and then saying*,* no*,* please don’t come now*,* because the wound care is about to start.*” (LU-10, male, 33 years). For some patients, it was not clear if the item referred to physical or rather mental burden induced by the treatment.

#### Item 6 (Unhappiness)

For some patients, the long healing process caused unhappiness, but mostly this was only temporary: “*Yes*,* every now and then you are in an emotional low because of the wound*,* then it just passes again*.” (DFU-01, male, 68 years). Unhappiness was also caused by restrictions in movement, in completing tasks and in vacation planning. Some patients were unhappy because they feared possible amputations.

#### Item 7 (Frustration)

Frustration was caused through setbacks in healing: “*Yes*,* because I always asked myself*,* does the toe have to be removed now*,* does it not have to be removed? Will it heal*,* will it not heal? Then there was an improvement again*,* then there was a deterioration again*,* that’s why you worry*,* right.*” (DFU-04, male, 62 years). For some patients, knowing that healing will take time reduced frustration, while specific personality traits caused frustration (“*Yes*,* I am impatient*,* so I am a bit frustrated that the healing takes so long.*” LU-08, female, 77 years). For some, it was frustrating when they felt that everything and the whole day revolved around their wound.

#### Item 8 (Worries)

The item on worries was relevant for all patients. They were primarily concerned about a worsening of the wound and prolonged healing: “*Yes*,* of course I’m worried. Because it used to be healed and now it won’t heal.*” (LU-07, female, 79 years). Also, patients worried about possible amputations or possible new wounds.

#### Item 9 (Worsening)

The fear of worsening was most often mentioned in relation to worsening of the present wound instead of new wounds appearing (“*When you suddenly see a small wound and it gets bigger and bigger*,* and then another pops up somewhere else*,* that’s when you get scared.”* DFU-02, male, 62 years). A worsening was sometimes also associated with potentially necessary amputations.

#### Item 10 (Knocking)

The item on knocking the wound was rated as relevant and irrelevant by the same number of participants. While some feared pain associated with knocking the wound or were concerned about possible deterioration of the wound (“*You always have the thought that you’ll bump into something or something*,* that the wounds will open up again.*”, LU-05, female, 54 years), others felt safe due to padding.

#### Item 11 (Moving)

In the item about moving, patients mainly thought about walking. This was considered as means of transport or as everyday activity: “*Walking. Walking. Because I don’t think the wound affects my other forms of moving about*,* such as driving a car or cycling or things like that. No*,* no*,* the focus is on moving as walking.*” (LU-04, male, 72 years). For some patients, impaired mobility led to reduced social contacts and increased risk of falling. Some mentioned that it was difficult to answer this item only referring to the wound as this aspect might also be impacted by other conditions: “*I can’t move anyway because I have problems with one knee (…). I have to rethink and then just think about the wound.*” (LU-08, female 77 years).

#### Item 12 (Climbing stairs)

Climbing stairs was discomforting for some patients, referring to climbing up and down *stairs: “Well*,* I can’t do that very well*,* but I can climb.*” (DFU-02, male, 62 years). Similarly to the item on moving, other conditions were also mentioned as impacting on climbing stairs. Almost as many patients rated this item as irrelevant compared to those rating this item as relevant.

#### Item 13 (Everyday activities)

Participants related everyday activities mostly to the household, but also to daily travels: “*Doing the laundry and a bit of housework*.” (LU-10, male, 33 years); “*I felt quite restricted*,* yes*,* I ticked the box here too*,* especially when it came to shopping.*” (DFU-08, male, 51 years).

#### Item 14 (Leisure activities)

Leisure activities were mainly associated with trips: “*Yes*,* for example*,* you can’t even go to a neighborhood with lots of places to eat and so on.*” (DFU-05, male, 67 years). Often, patients came up with activities in which water is involved, such as going to the swimming pool, sauna or the beach: “*That brings us back to the topic of going to the swimming pool or just doing something*,* yes*,* it often happens that I get to points where I say*,* hm*, sh*t,* you can’t do that now.*” (LU-03, male, 56 years).

#### Item 15 (Contact with others)

The item on contact with others was also associated with trips, but also with meetings or visits: “*That you meet up with friends*,* do something*,* so to speak.*” (DFU-01, male, 68 years). Few patients were uncertain if they comprehend the item correctly, e. g. as they thought about the time restrictions due to professional wound treatment. As activities with others also included sports, again overlapping with other conditions impacting on sporting activities needed to be considered.

#### Item 16 (Dependence)

Dependence on others was considered both regarding private support (“*So I’m dependent on the others. I have to ask someone to go with me. Then*,* when they go shopping*,* […] I’ve got everyone in the family going. Do you have to go there? Can you bring me this?*”, LU-05, female, 54 years) and regarding support from healthcare professionals. While the latter can be associated with negative feelings (“*YES*,* YES*,* I feel really dependent. I feel dependent. That’s stupid.*”, DFU-01, male, 68 years), but it can also be associated with positive feelings: “*It’s what it is. Well*,* in the past*,* you ran to anyone who sort of wanted to help you. Now I feel like I’m in really good hands here. It’s just that you have to get help. Otherwise*,* it wouldn’t work.*” (LU-01, female, 42 years).

#### Item 17 (Financial Burden)

As financial burden, most often co-payments for bandages and other medical products were considered. Some patients also considered travelling costs to physician visits or financial losses due to inability to work. Many participants considered the item as not relevant. However, some thought the item could be relevant for other patients.

### Relevance

Six patients commented on the relevance of the Wound-QoL in general. These statements emphasised the importance of this wound-specific questionnaire: “*So*,* these are all very important questions.*” (LU-02, male, 58 years); “*I know the questionnaire; I always like to fill it out because I have the feeling that all the practitioners draw their conclusions from it. That’s also important.*” (LU-06, male, 79 years). Few potential additional questions were mentioned: “*[Where I live]*,* you have to use public transport to get anywhere. Maybe that should be included somehow. You don’t usually drive a car anymore and public transport is limited.*” (LU-08, female, 77 years); “*And then maybe*,* do I have any questions?*” (LU-01, female, 42 years).

### Comprehensiveness

The majority of participants rated the scope of the Wound-QoL as appropriate and comprehensive. Patients with PU being treated in an inpatient setting rated specific items, such as everyday or leisure activities, as not relevant for the given context. Even though differences in the activities were reported item-wise with the think-aloud method, several patients suggested to connect the items on leisure activities and social contacts, partly also the item on everyday activities. Further items that were suggested to be connected were mostly items from the subscale psyche, partly also with the item on treatment burden. Some patients suggested connecting the items on moving and climbing stairs, and some stated that the item on sleep was covered by the item on pain.

### Comprehensibility

Participants rated the items in general as comprehensible (“*That is really sufficient and adequately presented and legible for every average consumer*,* it’s understandable*,* without technical terms.*”, DFU-06, male, 72 years). The instructions were clear for the participants (“*No*,* that’s all clearly and unambiguously formulated*.”, LU-07, female, 79 years), but some struggled with the term “chronic wound(s)” (German wording: “chronische Wunde(n)”) as they did not consider their own wound to be chronic due to their specific type of wound or due to the wound being currently closed. The response scale was considered suitable, though some patients suggested the use of smileys to make responding easier and more intuitive.

Regarding the recall period, some found 7 days appropriate as it would be difficult to reconstruct time spans going beyond a week, while others thought 10 to 14 days would also be appropriate. Some patients were concerned how episodes with severe burden could be depicted when not being within the recall period: “*Can we tick ‘not at all’ for this question; it says ‘in the last seven days’ at the top*,* then I would have to tick ‘not at all’ for the first question: Did I have pain in the wound? ‘not at all’*,* but when I think about it*,* the pain I had in the foot*,* in the open areas*,* was really in the range from ‘quite’ to ‘very’*,* so*,* um*,* it’s a bit difficult.*” (LU-10, male, 33 years).

### Wound-QoL-17 vs. Wound-QoL-14

Patients were explicitly asked about the three items that were deleted in the Wound-QoL-14, namely knocking, climbing stairs and financial burden. For all three items, some patients stated that they should be kept (knocking: “*So I think [item number] 10 makes a lot of sense*,* to be honest.*”, DFU-08, male, 51 years; climbing stairs: “*I’m very concerned about that when climbing stairs. I would take that into account.*”, LU-01, female, 42 years; financial burden: “*Can stay in*,* because it’s somehow true.*”, DFU-03, male, 56 years), while others stated that these items could be deleted (knocking: “*I don’t think knocking the wound is actually important for the medical procedure here. Whether I’m afraid of knocking the wound*”, LU-03, male, 56 years; climbing stairs: “*Climbing stairs is part of getting around. I think you could leave it out.*”, LU-03, male, 56 years; financial burden: “*Yes*,* it’s a financial burden for me*,* but it’s nobody’s business. It’s my private matter.*”, LU-02, male, 58 years).

### Further aspects

In addition to the deductively developed categories, patients came up with suggestions for improvement. This included to consider the specific situation of the patient, such as current treatment or healing process of the wound: “*I think they should differentiate between patients whose treatment has been completed and people who are still undergoing acute treatment*,* and then it should be formulated differently.*” (LU-07, female, 79 years), as well as to consider the impact of other conditions: “*So*,* secretly*,* I would like to see a little bit of an addition like that […]. You can have other illnesses that restrict your contact with friends or leisure activities or something like that.*” (LU-04, male, 72 years).

## Discussion

The aim of this study was to investigate the content validity of the Wound-QoL-17 and -14. Overall, the results show that the questionnaire is relevant, comprehensible and comprehensive for patients with chronic wounds.

Before discussing the results in more detail, it should be noted that this study was conducted in Germany and refers to the original German-language version of the questionnaire. Although linguistic adaptations of this questionnaire follow a well-established process that aims to maximize between-language similarity of content, interpretations and issues might slightly differ across language versions and countries.

Participants mostly understood the distinct items very well and thought they were easy to answer and relevant to their lived experience of having a chronic wound. The think-aloud method gave insights into what patients consider when answering each item. Often, the range of aspects being considered varied across individuals. For example, reading the term “treatment” (German wording: “Behandlung”), some patients only thought of treatment in hospitals and physician practices, whereas others also thought of wound care at home. In the development of the Wound-QoL, the aim was to develop a questionnaire that is easy to understand and both short and sufficiently comprehensive. This is why questions were framed specific enough for patients to understand them and to identify with the meaning, yet generic enough to be applicable to the entire patient population.

In some cases, patients considered partially overlapping content when answering different items. This was true, for example, for the item 1 on pain and item 4 on sleep. However, responses from patients showed that pain did not only disturb sleep but that there were other relevant aspects too, and that sleep could also be impacted by further aspects such as exudate and wound dressings. Therefore, it it is reasonable to keep both items distinct.

Similarities were seen between items from the psyche subscale. In particular, for item 6 on unhappiness, item 7 on frustration and item 8 on worries, prolonged healing was discussed as a possible cause. Despite these similarities, patients also explicitly differentiated between these emotions (e.g., “*rather frustrating than unhappy*”, DFU-08, male, 51 years / “*a bit annoyed*,* you can’t say unhappy*”, DFU-07, male, 42 years). Regarding statistical aspects of the questionnaire, we found that all items have different informative value for the specific subscale [32]; also, keeping multiple items with similar content may be beneficial as this can increase scale reliability. In clinical practice, being able to differentiate between different forms of mental impact might be meaningful as these may lead to different conclusions and therefore different support measures to be discussed. For example, while frustration due to slow healing could be addressed by taking time for explaining the disease course to be expected, worries could be addressed by identifying their causes and possibly referring for psychosocial counselling.

Some patients found it difficult to differentiate between item 14 on recreational activities and item 15 on contact with others. The proximity of these items was also seen statistically in a confirmatory factor analysis using international data, where correlations between these two items improved the models of both the Wound-QoL-17 and the Wound-QoL-14 [26]. As discussed above, items with similar contents are not necessarily undesirable as long as they are considered relevant and understandable by the patients (which was confirmed in the interviews). In a possible further shortened version of the questionnaire, combining these items could be considered. In view of the questionnaire already being short and the extensive psychometric data on the current item wording, benefits of a further shortened version would have to be weighed.

For some items, patients had difficulty distinguishing between HRQoL impairments caused by the wound and those caused by other issues. For example, this was the case for item 11 on moving and item 12 on climbing stairs. Chronic wounds are more frequent in older people [4], are mostly caused by an underlying disease [2], and are often associated with co-morbidities [8]. Therefore, many people with chronic wounds also experience other conditions that cause physical limitations. Nevertheless, many patients rated this item as relevant with regard to their wound. Additionally, the current wording is already as specific as possible explicitly stating “because of the wound”.

The item on odour shows that wounds and patient experiences are highly individual, highlighting the importance of measuring HRQoL. While some patients reported odour to be irrelevant for their own situation, none of them indicated that this item should be deleted from the questionnaire. For those patients rating this item as relevant, this was of particular importance. Also, previous studies show that malodorous wounds can largely impact patients’ social live and psychological state [33–36].

The Wound-QoL instructions and response scale were considered adequate and easy to understand. The idea to add smileys might further facilitate responding to the questionnaire for some patients.

Defining the recall period of a HRQoL questionnaire is a decisive task. While too short periods might lead to information loss and limited generalisability, longer periods might increase recall bias (i.e., memory bias) [37]. Patients were at odds if the recall period was adequate, with some arguing for a longer period, as seven days would not include times when their HRQoL burden was worse than at the time of completion. In contrast, regarding the item on pain, a number of patients had some difficulties answering this item even for a time span of seven days as pain is quite variable even within a single day. Therefore, the defined recall period of seven days might be assumed to be a reasonable compromise.

Some patients suggested taking the setting and patient characteristics into consideration, such as in- and outpatient treatment, time point in the healing process and age of the patient. In our study, patients completed the Wound-QoL-17 on paper, while patient characteristics were assessed verbally. In both the clinical context and in research, the above-mentioned patient characteristics should and usually will be recorded separately from the Wound-QoL (and possibly other PROMs). Therefore, an additional recording of these aspects within the Wound-QoL would be superfluous in many contexts.

The items on knocking the wound, on climbing stairs and on financial burden are deleted in the Wound-QoL-14 [24]. The number of patients rating them as relevant and those rating them as irrelevant were balanced, also when explicitly being asked whether these items should be deleted. The item on financial burden is a more distal aspect of quality of life that is highly influenced by the healthcare system instead of the treatment, but is still of importance to a share of patients. We therefore recommend to use the Wound-QoL-17 in clinical contexts, where a differentiated assessment of HRQoL is needed. In research, where the calculation of scores is paramount, we recommend using the Wound-QoL-14 as it has shown better psychometric properties [24–26].

This study fills a research gap as it is the first to investigate content validity of the Wound-QoL questionnaires. The strength of this study is that it was developed following the COSMIN Study Design Checklist for PROMs [38]. Conducting the interviews in-person at the clinics might have increased the participation rate, as patients could take part in the study during their routine visit at the clinics. Additionally, we assume that conducting the interviews in the clinical setting possibly made patients feel safer even with their highly visible and potentially odorous disease, despite the interviewer not being a clinical professional. Conversely, conducting the interviews via videoconference or telephone might have made some patient feel freer, as they would not have had to worry about the visibility of their wound (dressing) or odor. However, especially videoconferences might not be the most suitable method for this rather old study population, as it could pose a technical barrier to participate. A limitation is that while we recruited the targeted number of patients with LU and DFU, we were able to recruit only two patients with PU, despite promoting this study in a network of registered physicians in addition to the recruitment in two wards of a university hospital. Many patients with PU might be dependent on a wheelchair, be treated in intensive care or be bedridden, which makes it difficult to recruit these patients, as reported previously [39]. Therefore, we cannot make a statement about content validity of the Wound-QoL in patients with PU. This should be further investigated in future studies. Until then, use of a PU-specific HRQoL questionnaire might be considered, such as the PU-QOL [40].

## Conclusions

Our findings suggest that the Wound-QoL questionnaire has sufficient content validity for patients with LU or DFU and that it adequately reflects the construct of wound-specific HRQoL.

## Supplementary Information

Below is the link to the electronic supplementary material.

Supplementary Material 1: A1: Interview guide

## Data Availability

The datasets used and/or analysed during the current study are available from the corresponding author on reasonable request.

## Abbreviations

HRQoL: Health-related quality of life
FLQA-w: Freiburg Life Quality Assessment for Wounds
CWIS: Cardiff Wound Impact Schedule
WWS: Würzburg Wound Score
COSMIN: Consensus-based Standards for the selection of health Measurement Instruments
PROM: Patient-reported outcome measure
COREQ: Consolidated criteria for reporting qualitative research
LU: Leg ulcer
DFU: Diabetic foot ulcer
PU: Pressure ulcer
