# Supplementary material for: Content validation of the Wound-QoL questionnaire measuring quality of life in chronic wounds – a qualitative study in patients with leg ulcers and diabetic foot ulcers
Source: J Patient Rep Outcomes. 2025 Oct 22;9:124. doi: 10.1186/s41687-025-00935-9 (PMC12545981; doi:10.1186/s41687-025-00935-9)
Supplement: Supplementary file 1 — Supplementary Material 1: A1: Interview guide [file 41687_2025_935_MOESM1_ESM.pdf]

Wound-QoL - Interview guide

Thank you for agreeing to take part in the study.

Brief introduction of the interviewer.

With this project, we would like to find out whether the "Wound-QoL" patient questionnaire asks about all the important topics that have an impact on your quality of life as a person affected by a chronic wound. We also want to clarify whether the questions of the measurement instrument, as well as the instructions and answer options, have been formulated in a clearly understandable way.

The results serve to confirm the "Wound-QoL" questionnaire, which has been used in science and clinical practice since 2014, in its current form or, if necessary, to suggest a revision of the content.

We look forward to hearing your personal opinion and getting to know your views; there is no right or wrong in everything you say. I will keep my own part of the conversation to a minimum so that we can learn more about you and from you.

I will record our conversation and it will then be written down verbatim. We will remove personal information such as your name or places that you may mention in the conversation so that no conclusions can be drawn about you personally. Apart from this pseudonymised data (e.g. verbatim quotes to substantiate a statement), none of the information you share with me will be shared with anyone outside the study team.

The interview will last about 30 minutes - if we are finished earlier, this is not a problem and is just as informative for us as a longer appointment. You can stop the interview at any time or take a break if you wish. You also have the option to withdraw your consent, in which case we will delete the recording.

Do you have any questions? Then I would start the recording now.

Questions	Notes
Introductory questions	
To start with, could you give me a brief overview of how long you have been involved in the topic of chronic wounds?	Wound care situation
How long has the current wound been present?	Type / location of wound
In the next part of the interview we will talk directly about the questionnaire, so please fill it out now and tell me everything that is on your mind as you answer the questions.	
COSMIN subject areas (comprehensibility, scope, relevance)	
Comprehensibility and relevance	
Are there any questions that you hesitated to answer or that you did not find clearly formulated because you had doubts about how the question might be meant?	Comprehensibility: Items: every question
How do you understand the content of the individual questions?	Comprehensibility: every question
How important is this question to you in terms of your perceived quality of life?	Relevance: every question
Is there anything that could be improved in the instructions of the questionnaire?	Comprehensibility: Instruction
Is there anything in the answer options "not at all" to "very much" that could be changed?	Comprehensibility: Scale
Should the period of seven days to which the questions refer be changed?	Comprehensibility: Memory space
Scope	
In your opinion, do the questions in the "Wound-QoL" cover all areas relating to quality of life, or are you personally missing one or more aspects?	
Are there any questions that you feel are superfluous?	
Which version of Wound-QoL do you think is better? (Version Wound-QoL-14 versus Wound-QoL-17)	
Conclusion	

Is there anything else you think is important that we haven't discussed yet?	

STOP RECORDING.

Thank you very much for taking part in the interview.

Finally, I would like to ask you for some general information about yourself.

- Age
- Gender
- Highest educational qualification
- Housing situation (accessibility of the home and social situation)

I would like to thank you very much for taking the time to conduct the interview with me and also for your openness during our conversation. These are very important findings for us, which will help us to evaluate the "Wound-QoL" questionnaire.
